# Hypertension as the Leading Cause of Stroke in Young Adults in a Northern Nigerian Hospital: A Retrospective Study

**DOI:** 10.7759/cureus.105849

**Published:** 2026-03-25

**Authors:** Somadila A Igboanugo, Deborah O Adeyemo, Oboghene S Olori, Zinat A Jimada

**Affiliations:** 1 Cardiology, Rostov State Medical University, Rostov-on-Don, RUS; 2 Internal Medicine, Federal Teaching Hospital Katsina, Katsina, NGA; 3 Surgery, Federal Medical Center, Keffi, NGA; 4 Anesthesiology, University of Uyo Teaching Hospital, Uyo, NGA

**Keywords:** africa, hemorrhagic stroke, hypertension, ischemic stroke, young adults

## Abstract

Background and aim: Stroke is traditionally considered a disease of older adults; however, its incidence among young adults aged 18-45 years is increasing globally. In sub-Saharan Africa, stroke burden remains high, yet data on its risk factors in young adults are limited. This study aimed to identify and determine the predominant risk factors for stroke among young adults in Northern Nigeria.

Methods: A hospital-based retrospective study was conducted at Federal Teaching Hospital Katsina. Medical records of patients aged 18-45 years with brain imaging-confirmed stroke from January 2023 to December 2025 were reviewed. Demographic and clinical data, including stroke subtype and risk factors, were extracted. Descriptive statistics summarized the distribution of risk factors and stroke subtypes. Age differences between stroke types were analyzed using the Mann-Whitney U test, and associations between risk factors and stroke subtype were assessed using Fisher’s exact test.

Results: A total of 36 young adults met the inclusion criteria. Females accounted for 21 (58.3%) cases, while males accounted for 15 (41.7%) cases. The mean age was 40.5±4.7 years for males and 35.3±7.7 years for females. Ischemic stroke was the predominant subtype, 25 (69.4%), with hemorrhagic stroke accounting for 11 (30.6%) cases. Hypertension was the most common risk factor, occurring in 19 (52.8%) cases and being more frequent in females than in males. Other risk factors included cardiac disease in three (8.3%) cases, diabetes mellitus in three (8.3%) cases, smoking in three (8.3%) cases, HIV infection in two (5.6%) cases, and illicit drug use in two (5.6%) cases. Most risk factors were restricted to one stroke subtype. No significant associations were found between individual risk factors and stroke subtypes (all p>0.05), likely due to the small sample size.

Conclusion: Hypertension is the predominant and clinically significant risk factor for stroke among young adults in Northern Nigeria, with ischemic stroke being the most common subtype. The predominance of modifiable risk factors underscores the need for early detection, routine blood pressure screening, lifestyle modification, and public health interventions targeting young adults. Larger multicenter studies are warranted to further characterize stroke epidemiology and guide preventive strategies in sub-Saharan Africa.

## Introduction

According to the World Health Organization (WHO), stroke can be defined as a rapidly developing clinical sign of focal or global disturbance of cerebral function, with symptoms lasting 24 h or longer or leading to death [1,2]. For decades, stroke has been known as a medical condition that affects mostly older adults, but nowadays, it is rising globally among younger adults between the ages of 18 and 45 years [3-5]. Stroke is an important cause of morbidity and mortality in young adults, and the socioeconomic impact it has is enormous [3]. Young adults constitute a significant proportion of the workforce, and when affected by stroke, the consequences extend beyond the individual to their families and the community at large.

There is a paucity of data to highlight the incidence and prevalence of stroke in young adults in Africa. The Stroke Investigation Research and Educational Network (SIREN) reviewed that sub-Saharan Africa has the highest burden of stroke worldwide, with age-standardized stroke incidence rates of about 316 per 100,000; prevalence rate of about 14 per 1,000 population, and one-month fatality rates of up to 40% [6]. In the cohort study done by SIREN, 24.3% patients with stroke were less than 50 years of age [6].

Stroke may present as an ischemic or hemorrhagic stroke, and a computed tomography or magnetic resonance imaging is usually required to determine the type of stroke that a patient is having [1,7]. Some clinical features that stroke patients present with include hemiplegia, tetraparesis, brisk reflexes, sensory deficits, vision impairment, hypophonia, difficulty in swallowing, right vocal fold paralysis, and palate hypotonia [6]. Any individual who has been noticed to have any of the aforementioned symptoms should be admitted to the hospital for proper management and subsequent follow-up after discharge.

The risk factors for stroke can be classified into modifiable and non-modifiable factors. The modifiable factors include hypertension, dyslipidemia, diabetes, HIV, sickle cell anemia, smoking, excessive alcohol intake, illicit drug use, oral contraceptive use, heart diseases, and migraine [7,8]. The non-modifiable risk factors that are attributed to stroke include age, sex (female), race (Black), genetics, and family history. The female gender has a certain risk factor that is related to their reproductive system, which is exposure to estrogen, and the longer life expectancy in this gender may also be a causative factor of stroke in them [4,6,9].

This study aimed to identify the risk factors for stroke among young adults aged 18-45 years in Northern Nigeria and to determine the predominant factor of stroke within this population. Increasing public awareness of these risk factors may encourage the development of targeted health policies to reduce the incidence of stroke in young adults. Furthermore, this research seeks to address the existing scarcity of data on stroke epidemiology in Africa.

## Materials and methods

This study was designed as a hospital-based retrospective study conducted at Federal Teaching Hospital Katsina, a hospital located in Northern Nigeria. The objective of this study was to identify the various risk factors and the most common risk factor of stroke in young adults in the facility. Medical records of all patients admitted and being managed for stroke were reviewed. Data were collected retrospectively from January 2023 to December 2025, a period during which the electronic health record (EHR) was used.

Sample size determination

**Figure 1:**
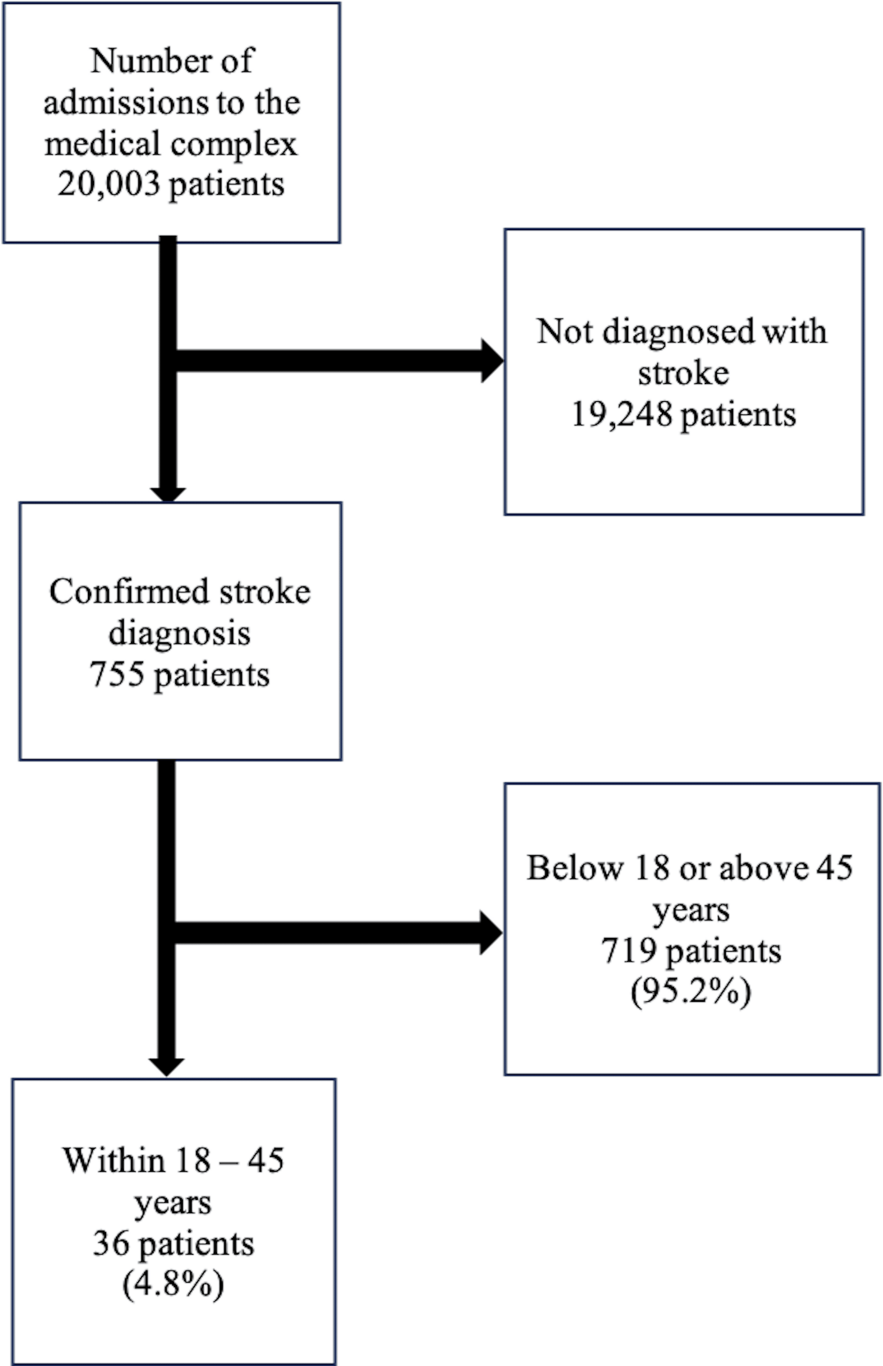
A diagram describing the inclusion and exclusion criteria.

No formal sample size calculation was performed because this is a retrospective descriptive study. All eligible patients aged 18-45 years who were diagnosed with stroke and met the inclusion criteria during the study period were included. Therefore, total population sampling was used. This is further highlighted in Figure 1.

Inclusion and exclusion criteria

Patients aged 18-45 years who were admitted with a diagnosis of stroke during the study period were included in this study. Individuals within this age group who presented with suspected stroke and had brain imaging (CT scan or MRI) confirming either ischemic or hemorrhagic stroke were also included. Additionally, only patients whose clinical records contained documented and identifiable stroke risk factors were considered eligible for inclusion. Patients younger than 18 years or older than 45 years were excluded from the study. In addition, patients within the specified age range without a confirmed diagnosis of stroke were also excluded.

Data collection techniques

Data were collected from the clinical records of patients who met the inclusion criteria. Demographic variables such as age and sex were extracted. Clinical information on the presence of risk factors, including history of hypertension, diabetes, sickle cell anemia, cardiovascular diseases, infectious diseases, alcohol intake, smoking, use of illicit drugs, use of oral contraceptives, pregnancy, cancer, autosomal dominant polycystic kidney disease (ADPKD), and obesity, was also obtained. Hypertension was defined as a documented history of high blood pressure, use of antihypertensive medications, or elevated blood pressure (≥140/90 mmHg) recorded at admission. Risk factors were obtained from clinical records and were clinically verified where documented. Multiple risk factors per patient were allowed and analyzed cumulatively. The type of stroke (ischemic or hemorrhagic), as determined by brain imaging, was also documented.

Statistical analysis

Data were entered and analyzed using Excel (Redmond, WA: Microsoft Corp.) and JASP statistical software (Amsterdam, Netherlands: JASP Team, University of Amsterdam). Demographic characteristics and stroke risk factors were summarized using frequencies and percentages only. Continuous variables, such as age, were expressed as mean±standard deviation (SD). In addition to descriptive analysis, exploratory inferential statistical tests were performed. Differences in age between ischemic and hemorrhagic stroke subtypes were assessed using the Mann-Whitney U test, while associations between categorical risk factors and stroke subtype were evaluated using Fisher’s exact test. Odds ratios (ORs) and 95% confidence intervals (CIs) were calculated to describe the direction and magnitude of these associations. Given the small sample size, all inferential findings were interpreted with caution and considered exploratory rather than confirmatory. A p<0.05 was considered statistically significant.

## Results

A total of 36 young adults aged 18-45 years with brain imaging-confirmed stroke were included. Females constituted the majority, accounting for 21 (58.3%) cases, while males accounted for 15 (41.7%) cases. The mean age of male patients was 40.5±4.7 years, whereas females had a lower mean age of 35.3±7.7 years, indicating greater age variability among female participants (Table 1). The mean age of patients with ischemic stroke was 37.6±7.2 years, compared with 37.1±7.1 years among those with hemorrhagic stroke. However, the difference in age distribution between the two stroke subtypes was not statistically significant (Mann-Whitney U=145; p=0.809) (Table 2). Ischemic stroke was the predominant subtype, observed in 25 patients (69.4%), while hemorrhagic stroke accounted for 11 patients (30.6%) (Table 3).

**Table 1:** Demographic characteristics and frequency of stroke risk factors. Data were expressed as mean±standard deviation or numbers (%) where appropriate. Descriptive statistics were used to summarize the data. Inferential statistical tests were applied where appropriate, as described in the methods section. ICSOL: intracranial space-occupying lesion; ADPKD: autosomal dominant polycystic kidney disease

Variables	Male	Female	Total, n=36 (%)
Age, years (mean±SD)	40.5±4.7	35.3±7.7	-
Sex, n (%)	15 (41.7%)	21 (58.3%)	-
Cardiac disease	-	3	3 (8.3%)
Hypertension	6	13	19 (52.8%)
Connective tissue disease	-	1	1 (2.8%)
HIV	-	2	2 (5.6%)
ICSOL	1	-	1 (2.8%)
ADPKD	1	-	1 (2.8%)
Smoking	3	-	3 (8.3%)
Diabetes mellitus	2	1	3 (8.3%)
Graves’ disease	-	1	1 (2.8%)
Illicit drug use	2	-	2 (5.6%)

**Table 2:** Comparison of patient age between ischemic and hemorrhagic stroke subtypes using the Mann-Whitney U test. Values are presented as mean±standard deviation (SD). Statistically significant p-value is <0.05.

Variable	Ischemic stroke (mean±SD)	Hemorrhagic stroke (mean±SD)	U	p-Value
Age (years)	37.6±7.2	37.1±7.1	145	0.809

**Table 3:** Association between clinical risk factors and stroke subtype (ischemic vs. hemorrhagic) using Fisher’s exact test. P<0.05 was statistically significant.

Risk factors	Stroke type		Odds ratio	95% CI	p-Value
	Ischemic	Hemorrhagic			
Cardiac disease	2	1	0.87	0.070-10.728	1.000
Hypertension	12	7	0.53	0.123-2.266	0.480
Connective tissue disease	1	-	1.40	0.053-37.288	1.000
HIV	2	-	2.45	0.108-55.257	1.000
ICSOL	1	-	1.41	0.053-37.288	1.000
ADPKD	1	-	1.41	0.053-37.288	1.000
Smoking	3	-	3.58	0.170-75.333	0.540
Diabetes mellitus	3	-	3.58	0.170-75.333	0.540
Graves’ disease	-	1	0.14	0.005-3.649	0.310
Illicit drug use	-	2	0.08	0.003-1.698	0.087
Total, n (%)	25 (69.4%)	11 (30.6%)	-	-	-

Hypertension was the most prevalent risk factor, identified in 19 patients (52.8%), with a higher proportion among females (13/21, 61.9%) than males (6/15, 40.0%). Cardiac disease was observed in three patients (8.3%), smoking and diabetes mellitus in three patients each (8.3%), HIV infection in two patients (5.6%), and illicit drug use in two patients (5.6%). Rare conditions, including connective tissue disease, intracranial space-occupying lesion (ICSOL), autosomal dominant polycystic kidney disease (ADPKD), and Graves’ disease, were each reported in a single patient (2.8%) (Table 1).

The association of risk factors with stroke subtype is summarized in Table 3. Hypertension was observed in both ischemic (12/25, 48.0%) and hemorrhagic (7/11, 63.6%) stroke cases. Cardiac disease affected two patients with ischemic stroke and one patient with hemorrhagic stroke. Smoking and diabetes mellitus were observed exclusively among ischemic stroke patients, whereas illicit drug use was reported only in patients with hemorrhagic stroke. HIV infection and rare conditions such as connective tissue disease, ICSOL, and ADPKD were associated exclusively with ischemic stroke.

Due to the small sample size, odds ratios (ORs) and 95% confidence intervals (CIs) suggested potential trends, rather than definitive associations. None of the associations reached statistical significance. For example, hypertension showed a non-significant association with stroke subtype (OR=0.53; 95% CI: 0.123-2.266; p=0.480), while smoking (OR=3.58; 95% CI: 0.170-75.333; p=0.540) and diabetes mellitus (OR=3.58; 95% CI: 0.170-75.333; p=0.540) were observed exclusively among ischemic stroke patients. Illicit drug use appeared more frequently among hemorrhagic stroke patients (OR=0.08; 95% CI: 0.003-1.698; p=0.087). Overall, hypertension remained the most common and clinically significant risk factor for both stroke subtypes. Other risk factors were less frequent and largely restricted to one subtype, reflecting the multifactorial etiology of stroke in young adults in this Northern Nigerian cohort.

## Discussion

This hospital-based retrospective study evaluated the risk factors and stroke subtypes among young adults aged 18-45 years in Northern Nigeria. Our findings demonstrate that hypertension is the predominant risk factor for stroke in this population, accounting for over half of all cases, i.e., 19 (52.8%) cases. In addition, ischemic stroke was more common than hemorrhagic stroke, representing 25 (69.4%) cases. These findings reinforce the growing concern that stroke is no longer confined to older adults and that modifiable cardiovascular risk factors are increasingly affecting younger populations [4,9].

Hypertension emerged as the most frequently observed risk factor and was associated with both ischemic and hemorrhagic stroke. This observation aligns with evidence from sub-Saharan Africa, including reports from the Stroke Investigation Research and Educational Network (SIREN), which identified hypertension as the most significant modifiable determinant of stroke in African populations [6]. Poor blood pressure control, late diagnosis, limited access to routine screening, and suboptimal adherence to antihypertensive therapy may contribute to this pattern in young adults [10,11]. The higher proportion of females in our study and the greater frequency of hypertension among them may reflect healthcare-seeking patterns, hormonal influences, pregnancy-related hypertensive disorders, or sociocultural factors that warrant further investigation [1,2,12,13].

Ischemic stroke was the predominant subtype in this cohort. Although hemorrhagic stroke is often reported to be relatively more frequent in African populations compared to high-income countries, recent trends suggest a rising burden of ischemic stroke among younger individuals due to increasing prevalence of vascular risk factors such as hypertension, diabetes mellitus, smoking, and cardiac disease [14-16]. In our study, most non-hypertensive risk factors, including diabetes mellitus, smoking, HIV infection, connective tissue disease, autosomal dominant polycystic kidney disease, and intracranial space-occupying lesions, were associated exclusively with ischemic stroke. This may reflect the thromboembolic and atherosclerotic mechanisms underlying these conditions [14,17].

Cardiac disease was identified in a notable proportion of patients and was associated with both stroke subtypes. Cardioembolic phenomena are well-established causes of ischemic stroke in young adults, particularly in the presence of structural or valvular heart disease [7,8,18]. Similarly, illicit drug use, which was associated with hemorrhagic stroke in this study, is a recognized precipitant of acute blood pressure surges and vascular injury, increasing the risk of intracranial hemorrhage [16,19].

The identification of HIV infection among some patients highlights the evolving epidemiology of stroke in sub-Saharan Africa. HIV-associated vasculopathy, chronic inflammation, and opportunistic infections are known contributors to cerebrovascular disease in younger individuals [3,6]. Although the proportion was small in our cohort, it highlights the importance of comprehensive cardiovascular risk assessment in patients living with HIV.

Although none of the associations between individual risk factors and stroke subtype reached statistical significance, the predominance of modifiable risk factors in this study emphasizes the critical need for early detection, preventive screening, and aggressive risk factor control among young adults. Community-based blood pressure screening programs, public health education campaigns, and improved access to primary healthcare services could significantly reduce the burden of stroke in this economically productive age group [20]. Given that young adults represent a substantial portion of the workforce, the socioeconomic consequences of stroke, including long-term disability and loss of productivity, are significant [21,22].

This study contributes valuable regional data to the limited literature on stroke epidemiology among young adults in Northern Nigeria. Overall, our findings reinforce the urgent need to prioritize hypertension control strategies among young adults in Nigeria. Addressing modifiable cardiovascular risk factors at earlier stages of life may substantially reduce stroke incidence, improve long-term outcomes, and lessen the socioeconomic burden associated with stroke in sub-Saharan Africa.

Limitations

The study’s retrospective, single-center design limits the generalizability of the findings to other regions in Nigeria or to sub-Saharan Africa. Reliance on electronic health records may have resulted in incomplete documentation of certain risk factors, potentially underestimating their prevalence. Additionally, the small sample size limited the power to detect statistically significant associations between risk factors and stroke subtype. No formal sample size calculation was performed, and the possibility of incomplete case capture or referral bias cannot be excluded, as more severe or complex cases may have been preferentially referred to this tertiary center. Despite these limitations, the study provides valuable regional data, with all findings clearly summarized in tables, contributing to the scarce literature on stroke among young adults in Northern Nigeria.

## Conclusions

This study demonstrates that hypertension is the predominant and most significant risk factor for stroke among young adults aged 18-45 years in Northern Nigeria. Ischemic stroke was the most common subtype, although hemorrhagic stroke remains a substantial contributor. However, due to the small sample size and retrospective design, these findings should be interpreted cautiously and viewed as observational trends rather than definitive conclusions. The predominance of modifiable risk factors highlights the urgent need for early detection, routine blood pressure screening, aggressive risk factor management, and public health interventions targeted at younger populations. Strengthening primary healthcare systems, improving awareness of cardiovascular risk factors, and promoting lifestyle modification strategies may significantly reduce the burden of stroke in this economically productive age group. Further large-scale, multicenter prospective studies are recommended to better characterize stroke epidemiology and guide preventive strategies in Nigeria and sub-Saharan Africa.
